# Development of Electroactive and Anaerobic Ammonium-Oxidizing (Anammox) Biofilms from Digestate in Microbial Fuel Cells

**DOI:** 10.1155/2015/351014

**Published:** 2015-07-27

**Authors:** Enea Gino Di Domenico, Gianluca Petroni, Daniele Mancini, Alberto Geri, Luca Di Palma, Fiorentina Ascenzioni

**Affiliations:** ^1^Pasteur Institute-Cenci Bolognetti Foundation, Department of Biology and Biotechnology “C. Darwin”, Sapienza University of Rome, 00185 Rome, Italy; ^2^Astronautic Electric and Energetic Engineering Department, Sapienza University of Rome, 00184 Rome, Italy; ^3^Department of Chemical Engineering Materials Environment, Sapienza University of Rome, 00184 Rome, Italy

## Abstract

Microbial Fuel cells (MFCs) have been proposed for nutrient removal and energy recovery from different wastes. In this study the anaerobic digestate was used to feed H-type MFC reactors, one with a graphite anode preconditioned with *Geobacter sulfurreducens* and the other with an unconditioned graphite anode. The data demonstrate that the digestate acts as a carbon source, and even in the absence of anode preconditioning, electroactive bacteria colonise the anodic chamber, producing a maximum power density of 172.2 mW/m^2^. The carbon content was also reduced by up to 60%, while anaerobic ammonium oxidation (anammox) bacteria, which were found in the anodic compartment of the reactors, contributed to nitrogen removal from the digestate. Overall, these results demonstrate that MFCs can be used to recover anammox bacteria from natural sources, and it may represent a promising bioremediation unit in anaerobic digestor plants for the simultaneous nitrogen removal and electricity generation using digestate as substrate.

## 1. Introduction

Anaerobic digestion (AD) of organic waste and/or animal manure is considered a key technology that meets the production of renewable energy with greenhouse gas mitigation. AD is accomplished through a series of complex microorganisms-driven reactions breaking down organic substances into CO_2_ and volatile fatty acids (acidogenesis) that are then converted to biogas (methanogenesis). The remaining fraction in the digester is a nutrient-rich sludge, the digestate. Although transformation of nitrogen compounds occurs in AD, the nitrogen content remains high in the digestate making this by-product suitable to be used as fertilizer. However, accumulation of biogas plants in small agriculture area or regions of intensive dairy cattle farming may lead to an oversupply of digestate. Indeed, digestate-based fertilization, when exceeding the need of crops, contributes to eutrophication of land and water bodies [1, 2].

One of the most promising techniques to reduce nitrogen content from ammonia-rich wastewaters is the anaerobic ammonium oxidation (ANAMMOX). Nitrogen removal under anaerobic conditions is driven by a group of anammox bacteria, which are affiliated to the order* Brocadiales*, within the phylum of* Planctomycetes* [3]. The advantages of the anammox process, over the conventional method of nitrification and denitrification, include the lower oxygen demand and the absence of external carbon sources requirements. However, a critical aspect limiting the application of this process in large bioreactors is the requirement of long start-up periods caused by the slow growth rate (doubling time approximately 1-2 weeks) of the anammox bacteria and by the absence of conventional microbiological techniques for their cultivation [4]. Several methods have been proposed to obtain enrichment of anammox bacteria [4–9]; however, cultivation still poses a serious challenge.

Nitrogen removal from wastewater has been studied in microbial fuel cells (MFCs), electrochemical devices that catalyse the conversion of chemical oxygen demand into electricity through the metabolic activity of microorganisms [10]. Typically in the anodic chamber, microorganisms oxidize the organic matter and the electrons are donated to the anode. These electrons are subsequently transferred, through an electric circuit, to the cathode electrode where they reduce terminal electron acceptors.

Several bacteria have naturally evolved strategies to transfer electrons outside the cell surface and this feature has allowed the use of these microorganisms in MFCs. The main quality of electroactive bacteria in the MFC system is the ability to transfer electrons from the microbial cell to an electrode instead of the natural redox partner [11]. Different microorganisms, either gram-positive or gram-negative, can exchange electrons with electrodes and this is accomplished by different mechanisms: reduction of self-produced soluble shuttles; short-range electron transfer through membrane-bound redox-active proteins (i.e., c-type cytochrome); long-range electron transfer mediated by a special class of conductive pili, the nanowires [12]. In most cases, bacteria may use more than one mechanism. For example, in* Shewanella oneidensis* the electrons may hop from the cell-surface* c*-type cytochrome, which is part of a multiprotein complex that transferred the electrons from the periplasm to the cell surface, to an external acceptor directly or via a flavin produced by the cell itself.* Geobacter sulfurreducens* has many c-type cytochromes exposed to the cell surface among which OmcZ appears to be the key element for electron transfer. Additionally, the conductive pili ensure the long-range electron transfer between the typical multilayer* G. sulfurreducens* biofilms and the electrodes.* G. sulfurreducens* current production is mediated by biofilms with a two-phase process in which long-range electron transport occurs along the conductive pili network and OmcZ facilitates electron transfer from those cells closer to the electrode surface [12]. However, when MFCs are inoculated with a mixed culture, bacterial community analysis of the anodic biofilms revealed a great diversity in electroactive bacteria, regardless of the substrate type. This finding suggests a potential existence of other unknown species contributing to electricity generation through a variety of ways beyond the accepted* Geobacter* or* Shewanella* species [13].

In addition to electricity production, the microbial metabolism can be used to produce valuable products or to remove unwanted compounds [14]. Accordingly, in the development of MFC, nitrogen removal has been considered as an added endpoint. Nitrate reduction at the cathode of MFC has been demonstrated by different experimental approaches including the use of a potentiostat-poised half-cell in which nitrogen was completely reduced to N_2_ gas in the absence of any organic substance (electron donors) [15]. More recently, a variety of denitrifying MFC reactors have been designed: two-chamber MFC reactors including both nitrification and denitrification steps at the cathode were obtained by aerated cathode chamber [16] or by preaerated cathode influent [17, 18]. Single-chamber MFC, with the cathode exposed to air (air-cathode), and two-chamber MFC, with ferricyanide catholyte were also tested for ammonia removal from swine wastewater [19, 20]. In both reactors electricity generations and high levels of ammonium removal were achieved; however, ammonia volatilization and/or diffusion through the cation exchange membrane connecting the anode and cathode chambers accounts for most of the nitrogen reduction [20]. More recently, single-chamber MFC reactors with PTFE (polytetrafluoroethylene) coated cathode, which reduces ammonia diffusion, and preenriched nitrifying biofilms were shown to remove up to 96% ammonia from a synthetic feeding medium [21]. Ammonia and COD removal rates by single-chamber MFC were significantly improved by doubling the gas diffusion area [22].

In this study we investigated the possibility of biological ammonia removal with current generation in MFC reactors from digestate. This was accomplished by feeding MFC reactors in batch mode with anaerobic digestate from agricultural by-products and cow manure. The reactors were H-type MFCs with a sterile graphite anode (MFC-U) and with a* G. sulfurreducens* preconditioned anode (MFC-C).* G. sulfurreducens* preconditioning was included as a control because of the well-known ability of this bacterium to convert organic matter into electricity in MFC devices [23]. Electrical and chemical performances of both cells were investigated. Additionally, the presence of anammox bacteria in the digestate and their establishment in the MFC conditions had been assessed by molecular methods. Our results demonstrate that MFC reactors allow the development of digestate-derived biofilms that contribute to the simultaneous generation of electricity and nitrogen removal from the digestate. However, since the substrate influences the bacterial diversity in the anode biofilm additional studies are needed to compare different types of digestate as well as to better understand the physical and biological mechanisms that can affect MFC performances in full-scale application systems.

## 2. Materials and Methods

### 2.1. Feed and Inoculum

The digestate was the effluent of an anaerobic digestion plant treating agricultural wastes and cow manure (Azienda Agricola Bruni, Sutri, VT, Italy). It was representative of a typical effluent of this kind of power plant in Italy [24]. The liquid phase of the digestate (pH = 7.98, Total Kjeldahl Nitrogen (TKN) = 4.9 g/L of N, total COD = 22.4 g/L), used for the experiments, was obtained by sieving the granules with a mesh of 2.36 mm pore size. Aliquots of 1 L were stored at 4°C and used during the first one-month feeding, frozen aliquots were used from the second month on.

### 2.2. Reactor Configuration and Operation

All experiments were performed using H-type MFC. The MFCs were done by two glass bottles (250 mL each) connected by a glass tube and a Nafion 117 proton exchange membrane (Sigma, UK) 7 cm^2^ in area, held by a clamp. Graphite electrodes (2.5 cm × 5 cm × 0.05 cm Goodfellow Cambridge Ltd, UK) were positioned 4 cm far from either side of the membrane. Each chamber was filled to 200 mL with feed solution in the anode and potassium phosphate buffer (100 mM pH 7.5) containing 50 mM K_3_Fe(CN)_6_ in the cathode. Anodic chambers were flushed with N_2_ gas to maintain anaerobic conditions. MFCs operated at constant temperature of 30°C in batch mode with a fixed external resistance, *R*_*ext*_, of 180 Ω. In the anodic chamber 150 mL of medium was replaced every 7 days leaving 50 mL of anolyte with each substitution. 200 mL of catholyte was replaced weekly. Feed solution (digestate-based feeding) was 1 : 10 digestate to medium; medium contained (per liter) KCl, 0.1 g; NH_4_Cl, 1.5 g; NaH_2_PO_4_, 0.6 g; NaHCO_3_, 2.5 g; Na-acetate, 0.82 g; vitamin solution 10 mL (Sigma-Aldrich), trace element solution, 10 mL [25]. Two MFC reactors operated under the same condition and configuration except the anode: MFC-U was assembled with a sterile anode; MFC-C was assembled with a precolonized* G. sulfurreducens* bioanode.* G. sulfurreducens* bioanode was obtained by a H-type MFC inoculated with* G. sulfurreducens* pure cultures.* G. sulfurreducens* was obtained from the German Collection of Microorganisms and Cell Cultures (DSMZ, Braunschweig, Germany).

### 2.3. Electrochemical Measurements

Open circuit voltage (OCV) and closed circuit voltage (CCV) across the external resistance (*R*_*ext*_ = 180 *Ω*) were monitored at 30 min intervals using a multichannel potentiostat/galvanostat VSP (Biologic Sas) connected to a personal computer. Current (*I*) was measured using the same instrument in a chronoamperometric mode, and Coulombic Efficiency (CE) was calculated as

(1) $$\begin{array}{c} \text{CE}\left(\%\right)=100\cdot \frac{C_{p}}{C_{\text{th}}}, \end{array}$$

where *C*_*p*_ was the total electric charge calculated by integrating the current over time and *C*_th_ was the theoretical amount of electric charge available based on the total COD removal in the MFC section of the reactor [26].

Power, calculated as

(2) $$\begin{array}{c} P=\text{CCV}\times I=R_{\mathrm{ext}}\times I^{2}=\frac{{\text{CCV}}^{2}}{R_{\mathrm{ext}}}, \end{array}$$

was normalized with respect to both the projected surface area of the cathode (power density) and the volume of the liquid media (volumetric power density). MFC internal resistance was estimated using both maximum power transfer theorem (varying the external load by a resistance box ranging from 0 Ω, that is, the short circuit condition, up to 4 kΩ. The internal resistance value coincides with the value of the external resistance that maximizes load power consumption) and drawing typical polarization curves (by using the VSP instrument).

### 2.4. Chemical Analyses

The pH was measured using a GPL 42 instrument (Crison). The dissolved oxygen was measured with a 913 OXY oximeter (Mettler-Toledo). The total COD was determined by acid digestion and dichromate titration, according to standard methods (APHA, AWWA, and WEF, 2005). In order to evaluate the TKN, sample was heated at 400°C after mixing with 98% sulphuric acid and K_2_SO_4_. The obtained solution was cooled, blended with NaOH, and distilled using the Kjeldahl method. The amount of nitrogen into the distilled solution was determined by spectrophotometry with the Nessler reagent, using a T80+ UV/Vis spectrometer (PG Instruments, Ltd). Nitrites and nitrates were determined by ion chromatography, using a DX 120 instrument (Dionex).

### 2.5. Microbial Community Analysis

#### 2.5.1. DNA Extraction and PCR Amplification

At the end of the experimental procedure (day 49) the graphite anode was cut into three sections. The surface of each slide covered by the anodic biofilm was scraped and placed in sterile 50 mM PBS; at the same time samples from the digestate and from different pure bacterial cultures were collected. Total DNA was extracted using the QIAamp DNA stool Mini-Kit (Qiagen Inc., Valencia, CA, USA) according to the manufacturer's instructions.

PCR amplification was carried out using the following primers: P0 and P6 for the bacterial 16S rRNA gene [27]; Geo587F/Geo978R targeting* G. sulfurreducens* and other closely related* Geobacter* [28]; amoA-1F/amoA2R targeting the AmoA gene that codifies ammonia monooxygenase (AMO) of ammonia oxidizing bacteria [29]; Brod541F-Brod1260R targeting the 16S rRNA gene specific for anammox bacteria [30]; and AnnirS379F-AnnirS821R targeting nitrite reductase gene of the anammox bacteria [31].

Real-time PCR was used to determine the relative abundance of the 16S rRNA gene of anammox bacteria in the MFC cultures with respect to digestate. First, the relative abundance of the anammox 16S rRNA gene with respect to total 16S rRNA gene (ΔCt) was determined for each sample (digestate, MFC-C and MFC-U); next the relative quantification (RQ) of the anammox gene in the MFC-C and MFC-U with respect to the digestate was calculated using the delta delta Ct method (2^−ΔΔ*Ct*^). SYBR Green real-time PCR assays were performed using the following primer sets: anammox 16S rRNA gene, AMX818F-AMX1066R described in Tsushima et al. 2007 [32]; universal 16S rRNA gene, U16SRT-F-U16SRT-R, designed in the consensus sequence of bacterial 16S rRNA gene [33]. All primer sets were tested for sensitivity, optimal annealing, temperature, and primer efficiency with proper positive and negative controls. The positive control for the anammox 16S rRNA gene amplification was a plasmid containing the sequence of the anammox 16S rRNA gene obtained in this study.

#### 2.5.2. Cloning and Sequencing of the 16S rRNA Gene

Brod541F-Brod1260R primers were used to amplify the 16S rRNA gene of the anammox bacteria. PCR products were purified from preparative agarose gels and cloned in the TOPO TA cloning kit (Invitrogen) for sequencing. The 16S rRNA gene sequences were compared for similarities to DNA sequences in the NCBI databases by BLAST. The phylogenetic tree was obtained using the multiple alignment program for amino acid or nucleotide sequences (MAFFT version 7). The sequences were deposited in the European Nucleotide Archive (ENA) with accession numbers LN714795-LN714796.

#### 2.5.3. Biofilm Imaging

Anodic biofilm samples were collected from each reactor by slicing 1 cm^2^ carbon anode with sterilized scissors in an anaerobic chamber. Samples were stained using the LIVE/DEAD BacLight kit (Invitrogen), according to supplier specifications and examined with Apotome Fluorescence Microscope (Carl Zeiss International). Data were collected and analysed with the Axiovision 4.8 software. Samples for SEM analysis were fixed in 2.5% glutaraldehyde (Sigma-Aldrich) in PBS solution (0.1 M, pH = 7.4) for 3 hours at 4°C, washed three times in the same buffer (10 min each), and then postfixed with osmium tetroxide solution (1% in 0.1 M phosphate buffer, pH = 7.2). After rinsing in phosphate buffer, the samples were dehydrated in a series of graded ethanol and air-dried. All samples were coated with a 10 nm thick gold film. Coated samples were examined using an electron acceleration voltage of 20 keV, in both the secondary and the backscattered electron modes using a LEO 1450 VP microscope.

## 3. Results and Discussion

### 3.1. Start-Up of the MFC Reactor Fed with Digestate

**Figure 1:**
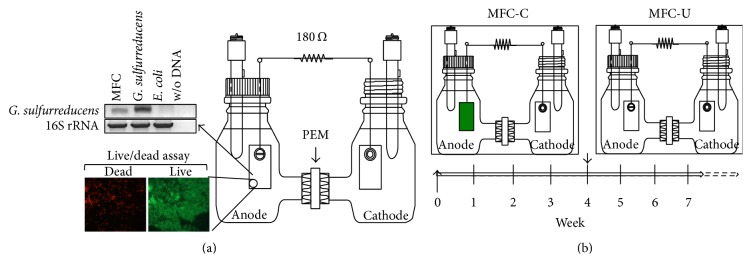
Schematic representation of the MFC-C and MFC-U reactors and the start-up phase. (a) H-type MFC inoculated with* G. sulfurreducens* and fed with synthetic medium containing acetate; after one-month operation small pieces of the anode were cut out and bacterial biofilm was visualized by the live-dead assay. Additionally, DNA samples were obtained from biofilm and amplified with universal or* G. sulfurreducens* 16S rRNA primers.* E. coli* and* G. sulfurreducens* DNAs were used as controls. (b) MFC-C was assembled with the anode from the reactor in panel (a); MFC-U was assembled with a sterile anode. Both reactors were operated under the same conditions and fed with digestate medium once a week. The arrowhead marks the transition from inactive to active MFC-U reactor.

**Figure 2:**
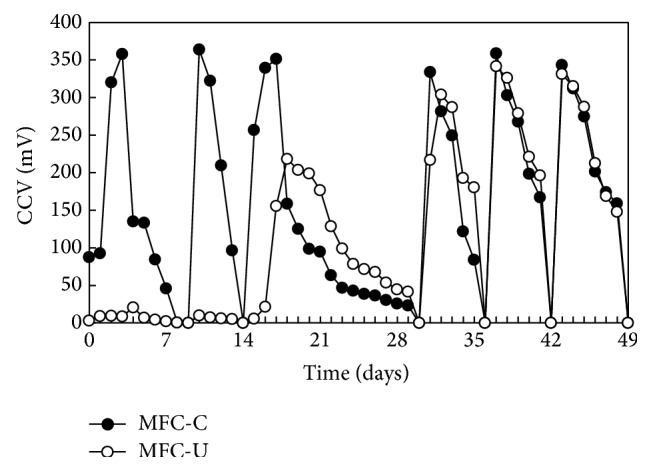
Voltage generation with digestate-feeding in MFC-C and MFC-U. Time 0 was the third week of operation.

In order to determine whether the resident microbial community of the digestate can convert organic matter into electricity while reducing nitrogen content we set up H-type MFCs with the two-chamber separated by proton exchange membranes (Figure 1). The first reactor (MFC-U) was assembled with a sterile anode whereas the second one (MFC-C) was assembled with a preconditioned* G. sulfurreducens* biofilm on the anode (Figure 1(b)). The latter was obtained from an MFC operated with* G. sulfurreducens* pure culture and synthetic feeding (Figure 1(a)). Both MFC-U and MFC-C were fed with a digestate-based medium as reported in materials and methods. The initial digestate was diluted in order to obtain more favourable conditions for the growth of the resident bacteria and to reduce the introduction of toxic compounds that may inhibit bacterial activity [34–36]. During the first month the reactors behaved differently: MFC-U did not reveal any cell voltage while MFC-C showed a rapid CCV increase after feeding (Figure 2). In the MFC-C, after each feed solution replacement, CCV increased reaching similar values as in the previous cycle. Additionally, the rapid increase of CCV observed following feeding strongly suggested that it was due to the activity of the anode-associated biofilm. In the first three weeks, after reaching the peak, the CCV decreased with a different rate, whereas from day 28 to the end of operation the CCV cycles were more homogeneous. This may be due to the presence of an evolving bacterial population in the anodic chamber that reached the equilibrium after 3-4 weeks of operation.

After the fourth refeeding the MFC-U showed an increase of the CCV to about half of that recorded in MFC-C and a subsequent decrease that appeared somewhat slower than that in the MFC-C. To synchronize the two reactors, the fifth feeding was postponed by one week, after which weekly feeding was restored. As expected, from day 28 on the MFC-U and MFC-C cycled similarly showed a rapid increase of CCV after feeding and subsequent decrease in the following 5-6 days.

After the start-up period MFC-C reached a reproducible maximum power (computed by 2) of 0.6 mW (i.e., 240 mW/m^2^) at 346.8 mV, similarly MFC-U reached a maximum power transfer of 0.4 mW (i.e., 172.2 mW/m^2^) at 359.4 mV. The time to achieve the maximum cell voltage after feeding (16–18 hr) was longer than that reported for MFC reactors fed with acetate [13] but similar to that obtained with slaughterhouse wastewater-fed reactors [13]. The substrate type influences the MFC performance, not only in terms of bacterial community but also in the maximum power and Coulombic Efficiency. Therefore, the time required to achieve the maximum cell voltage observed in our MFC systems is in accordance with the composition of the digestate-like medium supplemented with acetate.

It has been reported that methanogens, by competing with electroactive bacteria for substrates, can reduce the performance of MFCs [37]. Nonetheless, in both reactors we did not detect CH_4_ production, neither in the start-up period nor during operation regime, suggesting that methanogenesis did not take place in the MFCs or it was very low. Since the digestate was collected in the final stage of biogas production it may be that methanogenesis was exhausted although we cannot exclude that digestate-based medium in MFC conditions outcompeted methanogens while favoring colonization of electrogenic bacteria.

### 3.2. Electrochemical Performance

**Figure 3:**
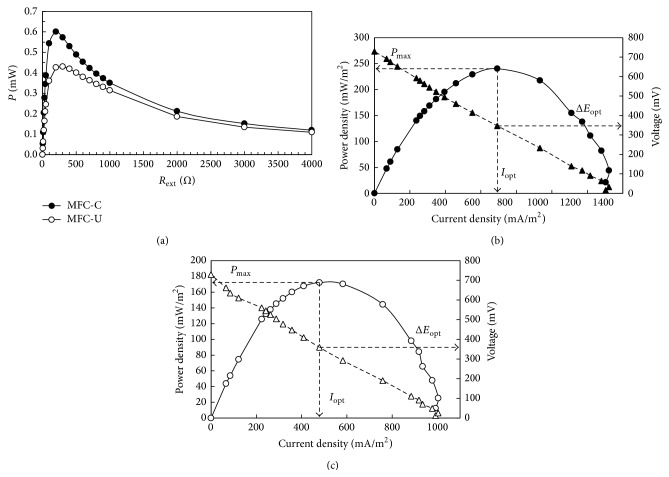
Maximum power transfer curves performed on MFC-C and MFC-U (a). Polarization curves with the maximum power density (*P*_max⁡_), the optimal voltage (Δ*E*_opt_), and optimal current density (*I*_opt_) performed on MFC-C (b) and MFC-U (c) at the end of the start-up procedure (day 28).

Maximum power transfer curves (Figure 3(a)) and polarization curves (Figures 3(b) and 3(c)) for both the MFCs were carried out in correspondence of CCV peak values at the sixth batch cycle when the reactors reached stable performances. The maximum power density was determined by varying the external resistance over a range of 0–4000 Ω and recording the voltage (Figure 3(a)).

The maximum power generation reached a peak value of 0.60 mW for the MFC-C and 0.43 mW for the MFC-U when the applied external resistance matched the internal resistance of the system at 200 and 300 Ω for the MFC-C and MFC-U, respectively.

The higher maximum power density and the reduced ohmic resistance of the MFC-C with respect to MFC-U might be ascribed to the* G. sulfurreducens* anode preconditioning. Conversely, in the MFC-U the digestate-resident microbial population might prefer slightly higher resistance conditions to better exploit the substrate as a result of the competition with the electrogenic bacteria.

The maximum power density per projected anode surface area was 170 mW/m^2^ for the MFC-C (Figure 3(b)) and 240 mW/m^2^ for the MFC-U (Figure 3(c)) while the limiting current density recorded was 1304 mA/m^2^ for the MFC-U and 992 mA/m^2^ for the MFC-C. Overall, the electrochemical measurements showed comparable performances between the reactors demonstrating that electrogenic bacteria were present in the digestate and possibly selected by MFC conditions.

### 3.3. Coulombic Efficiency and Substrates Removal

**Figure 4:**
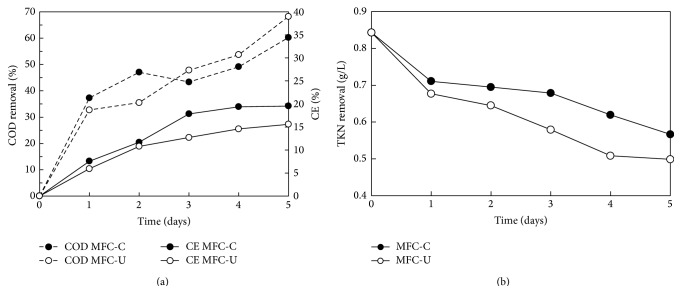
Total COD removal and Coulombic Efficiency (CE) in MFC-C and MFC-U (a); TKN removal as function of time (b) (days 36–41).

MFC-U and MFC-C exhibited during days 35 and 42, a calculated CE of 15.6 and 19.6%, respectively (Figure 4(a)), both of which were in the typical range observed for MFCs fed with waste or wastewater [19, 38] while CE, in MFC fed with acetate-based synthetic media, may rise up to 98% [13].

The absence of any gas development in both reactors allowed us to exclude methanogenic processes as the main cause of the resulting low Coulombic Efficiency. On the contrary, it could be attributed to the initial presence of nitrates, sulphates, and other terminal electron acceptors in the digestate.

The removal of the organic carbon by both reactors was quite efficient as demonstrated by the reduction of total COD up to 68 and 60% in the MFC-C and MFC-U, respectively, at the fifth day after feeding. Considering that the liquid digestate accounted for about 71% of the initial COD both reactors appeared to be very effective in the reduction of the organic content of the effluents from anaerobic digestion plants.

As regards to nitrogen TKN removals of about 40% and 32% in the MFC-C and MFC-U, respectively, were observed (Figure 4(b)). Ammonia in the cathodic chamber was always found at negligible level disfavouring the hypothesis of the ammonium ion transit to the cathodic chamber through the membrane [39]. Ammonia volatilization in the cathodic chamber can be also excluded since no oxygen was insufflated and the cathodic pH was neutral. Nitrogen sources in the MFCs were both the growth media (as ammonia) and the digestate (mainly as organic nitrogen and ammonia); the latter accounted for about 60% of nitrogen in each fed-batch cycle. Although, nitrogen consumption in the cells could be in part justified by the synthesis of new biomass in the anodic chamber, other mechanisms could be involved. According to the literature ammonia consumption in MFC can be also explained by several different specific pathways such as nitrification-denitrification, anammox, and nitrite reduction by lithotrophic ammonia oxidizers or by specific processes of ammonia oxidation coupled to electricity generation. Taking into consideration the lower Coulombic Efficiency and total COD consumption calculated for the unconditioned cell with respect to the conditioned one, electricity generation from direct ammonia oxidation appeared to be negligible. At the same time, since dissolved oxygen in the anodic chambers was always lower than 0.05 mg/L and that nitrates and nitrites at the end of each cycle were negligible in the anodic and cathodic chambers in both MFCs, it is reasonable to hypothesize that the main nitrogen removal mechanism was ammonia oxidation under anaerobic conditions.

Collectively our results suggest that H-type MFC reactors fed with digestate-based medium allowed the development of a microbial consortium able to oxidize ammonia anaerobically, as proposed in other studies [40]. However, further experiments are needed to better investigate the nitrogen removal mechanism and to evaluate the maximum nitrogen amount potentially degradable in such systems.

### 3.4. Biofilm Imaging

**Figure 5:**
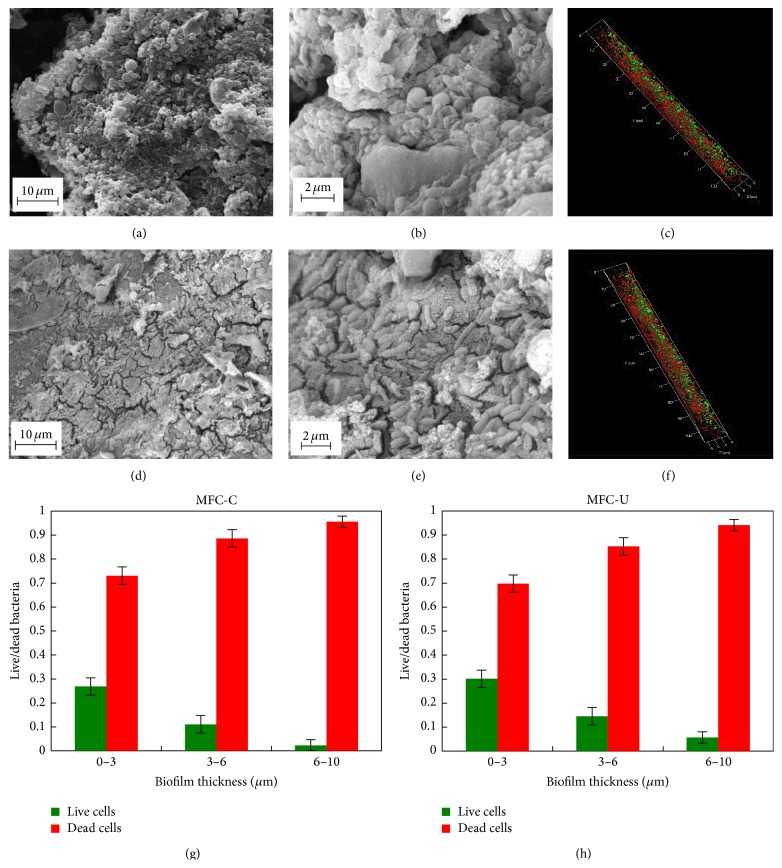
Biofilm imaging and cell viability. (a–f), SEM images of biofilms from MFC-C (a and b) and MFC-U (d and e). Live (green) and dead (red) bacteria within the biofilms from MFC-C (c) and MFC-U (f). (g and h) Fraction of live and dead bacteria in the anode biofilms from the indicated reactors. Cell viability was determined at the surface (0–3 *μ*m), in the middle (3–6 *μ*m), and at the base of the biofilms (6–10 *μ*m).

The morphology of the biofilm grown on the electrodes surface was analysed by scanning electron microscope (SEM) and fluorescence microscopy. Anode samples, about 1 cm^2^ size, were taken from the reactors operating since two and three months; the sampling was done the day after feeding when the reactors reached the maximum power generation. SEM analysis showed that anodes from both reactors were covered by bacterial biofilm (Figures 5(a) and 5(d)). Measurements of biofilm thickness showed that the biofilm ranged from 141 ± 30 *μ*m to 66 ± 1 *μ*m without detectable differences between MFC-C and -U. Close-up images (Figures 5(b)–5(e)) revealed a different bacterial morphology with a predominance of bacilli, often tightly embedded into the biofilm matrix (Figure 5(e)). Comparative analysis of the biofilms from the MFC-C and MFC-U anodes showed a more uniform morphology in the former than in the latter. Accordingly, MFC-C images at higher magnification revealed the presence of a multilayered biofilm in all the fields examined. On the contrary, MFC-U biofilm showed composite morphology with smooth and rough areas with bacteria mainly located on the surface of the matrix. Additionally, MFC-U biofilm showed the presence of complex aggregates, probably due to the entrapped digestate sediments. These different morphologies could be due to the fact that biofilm in MFC-C was previously colonized by* G. sulfurreducens* pure culture whereas MFC-U biofilm is developed on sterile anode by the unique contribution of the bacteria present in the digestate.

Next bacteria viability in biofilm samples was analysed using the LIVE/DEAD assay (Figures 5(c) and 5(f)). Fluorescence microscopy analysis did not reveal significant differences between MFC-C and MFC-U. 3D analysis showed that live bacteria preferentially localized on the outer layer of the biofilm (Figures 5(c) and 5(f)), probably due to easier availability to substrate. Quantitative analysis of the green and red signals within the biofilms showed that most of the live bacteria stratified in the outer layer and account for about 30% of total (Figures 5(g) and 5(h)). The fraction of live bacteria was slightly higher in MFC-U than in MFC-C, probably due to the fact that the anodic biofilm in the latter was one month older than the former. Although the staining procedure cannot rigorously distinguish live and dead cells, since it is based on membrane permeability, the fraction of live bacteria in these reactors appeared much lower than previously described [13]. Some differences may account for this result such as anode materials (graphite versus carbon cloth) and the age of the biofilm. Nonetheless, current generation in the two reactors was similar to that reported by the aforementioned studies, suggesting that the anode biofilms developed from digestate are efficient in electricity production. Additionally, the presence of a subpopulation of dead cells in-between the metabolically active cells and the electrode surface did not appear to significantly dampen electron transfer possibly due to long-range electrons transfer via the conductive pili [12]. We cannot exclude that the layer of dead cells may overgrow with time, reducing the efficiency of electron transfer to the anode. Although, the cycling in current production observed during the operation period suggests that the subpopulations of live and dead cells found equilibrium compatible with sufficient electron transfer.

### 3.5. Molecular Analysis of Biofilms

MFC-C and MFC-U performed very similarly although small differences in CE, nitrogen removal and biofilm structure were recorded, suggesting similar microbial communities developed on both conditioned and unconditioned anodes. To address this hypothesis, at the end of the last feeding cycle, DNA samples from both reactors were analysed by using the 16S rRNA gene as a molecular marker and functional biomarkers such as genes involved in the nitrogen metabolism. Controls DNAs were extracted from the same digestate used to feed the reactors, from* G. sulfurreducens* and laboratory strains of* Escherichia coli*. First, the 16S rRNA genes were amplified with universal primers and then restricted with* Rsa*I and* Hinf*I to analyse genome similarities between the two reactors and control DNAs (see Supplementary Material available online at http://dx.doi.org/10.1155/2015/351014) (Supplementary Figure  1)). The resulting restriction profiles showed high similarities among digestate, MFC-C, and MFC-U samples and between the MFCs and* G. sulfurreducens*.

**Figure 6:**
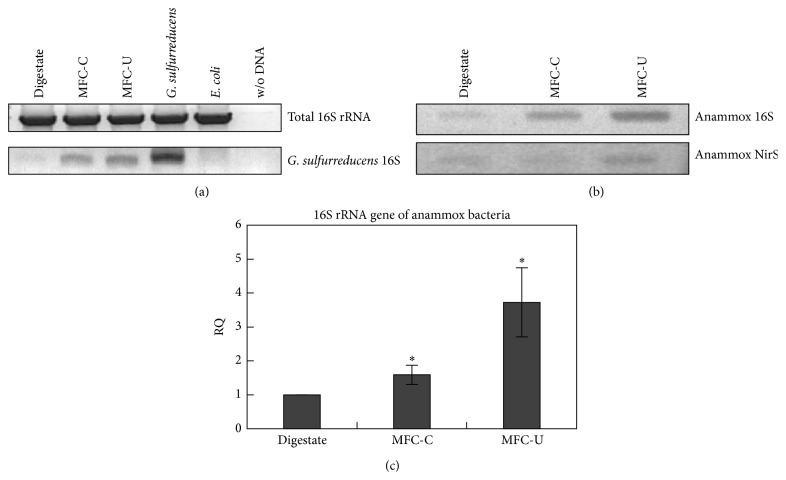
Molecular analysis of the biofilm. (a) PCR amplification of the indicated genes. 100 ng of DNA is used for each PCR reaction. (b) Upper panel, PCR amplification of the 16 rRNA gene, and the cd1 nitrite reductase (NirS) of the anammox bacteria. (c) Relative quantification (RQ) of the anammox 16S rRNA gene in the MFC-C and MFC-U biofilms with respect to digestate. Data are mean, standard deviation, *N* = 3; statistical analysis (*t*-test): MFC-C versus digestate *P* = 0.018; MFC-U versus digestate *P* = 0.011.

Next, 16S rRNA gene was also amplified by using specie-specific primers.* G. sulfurreducens* 16S rRNA gene was clearly detected in MFCs and digestate and the band intensity was higher in MFC-C than in MFC-U due to the* G. sulfurreducens* preconditioning (Figure 6(a)). Sequence analysis confirmed the presence of* G. sulfurreducens* in the digestate and in both reactors. No AmoA band targeting the ammonia oxidizing bacteria (AOB) [29] was observed in the reactors. This suggests that the anaerobic conditions of the anodic chamber, the accumulation of toxic compounds in the MFC, or the competition with better-adapted microorganisms negatively affected the growth of the AOB. On the contrary, by using primers targeting the 16S rRNA gene and the cd1 nitrite reductase (NIRS) of the anammox bacteria [41] (Figure 6(b)) we observed amplification products in both the reactors and the digestate. To quantify the abundance of anammox 16S rRNA in the bacterial populations of both reactors, we performed real-time PCR assays by using the relative quantification method and the digestate as calibrator (Figure 6(c)). The analysis showed an increase of the average abundance of the anammox specific 16S rRNA genes in the MFC-C (1.59 ± 0.28) and MFC-U (3.73 ± 1.01) compared with the digestate. This suggests a greater ability of anammox bacteria to colonize sterile MFCs than* G. sulfurreducens*-conditioned reactors.

Finally, sequencing analysis of the anammox 16S rRNA gene confirmed presence of* Planctomycetes* closely related to* Candidatus brocadia anammoxidans* (sequence identity between 97-98%) in both reactors (Supplementary Figure  2).

Overall, the molecular analyses revealed that MFC bacterial communities were directly related to the microbial population found in the digestate; irrespective of* G. sulfurreducens* preconditioning, very similar microbial communities developed in the MFC-C and MFC-U reactors; MFC operating conditions selected electrogenic bacterial and provide favourable conditions for the cultivation of anammox bacteria.

## 4. Conclusions

Two-chamber MFC reactors fed with anaerobic digestate and operated in batch-mode were assembled to test for simultaneous nitrogen reductions and energy recovery. Appreciable removal of total COD (up to 60%) and TKN (up to 40%), together with good electricity generation, was achieved by the activity of bacterial consortia derived from digestate. Regardless of preacclimation of the anodic biofilm with* G. sulfurreducens* in one of the cells, the proposed MFCs allowed the development of biofilms containing anammox bacteria in the anaerobic compartment of the MFC, indicating the presence of favourable conditions (e.g., strict anaerobic conditions and high nitrogen content) for these bacteria. The comparable current production measured in both MFC-C and MFC-U suggests that electrogenic bacteria, such as* G. sulfurreducens,* were fostered in the electrode colonization.

However additional studies are needed to better understand how the MFC environment and the digestate influence bacteria proliferation and biofilm development electricity generation and ultimately nitrogen removal. Additionally, in scaling-up MFC or in the assembly of continuous flow systems, the treatment of undiluted digestate could represent a critical issue especially at an acceptable hydraulic retention time. Nevertheless, the proposed results represent a preliminary study to address the feasibility of MFC as bottoming bioremediation units in anaerobic digestor plants to generate electricity and simultaneously treat digestate for nitrogen removal in order to limit waters pollution caused by spreading of livestock effluents.

## Supplementary Material

Supplementary Figure 1: Restriction analysis: electrophoretic analysis of the 16S rRNA genes after restriction with RsaI and HinfI enzymes for the reported samples.
Supplementary Figure 2: Phylogenetic tree: Unrooted neighbour joining tree based on the partial 16S rRNA gene sequences of the anammox bacteria from MFC-C and MFC-U. 16S rRNA of the anammox bacteria is amplified by a primer set of Brod541F-Brod1260R.
