# Supplementary material for: Development of Electroactive and Anaerobic Ammonium-Oxidizing (Anammox) Biofilms from Digestate in Microbial Fuel Cells
Source: Biomed Res Int. 2015 Jul 27;2015:351014. doi: 10.1155/2015/351014 (PMC4530212; doi:10.1155/2015/351014)
Supplement: Supplementary file 1 — Supplementary Figure 1: Restriction analysis: electrophoretic analysis of the 16S rRNA genes after restriction with RsaI and HinfI enzymes for the reported samples. Supplementary Figure 2: Phylogenetic tree: Unrooted neighbour joining tree based on the partial 16S rRNA gene sequences of the anammox bacteria from MFC-C and MFC-U. 16S rRNA of the anammox bacteria is amplified by a primer set of Brod541F-Brod1260R. [file 351014.f1.pdf]

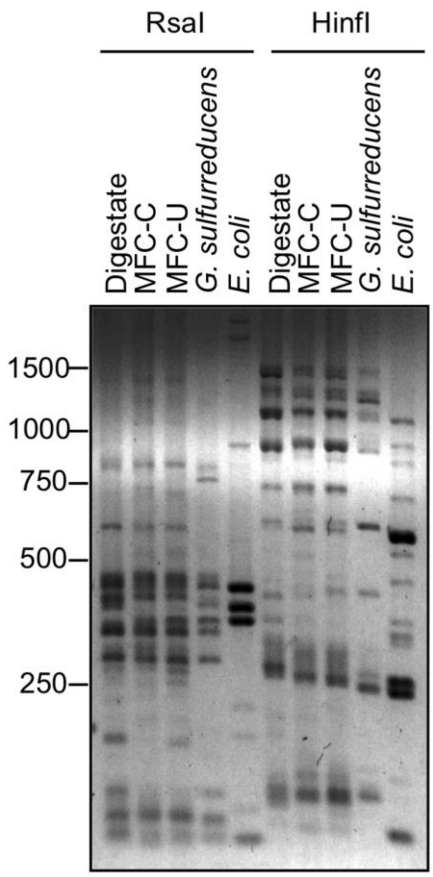

Supplementary Figure 1: Restriction analysis of the 16S rRNA genes with RsaI and HinfI for the reported samples

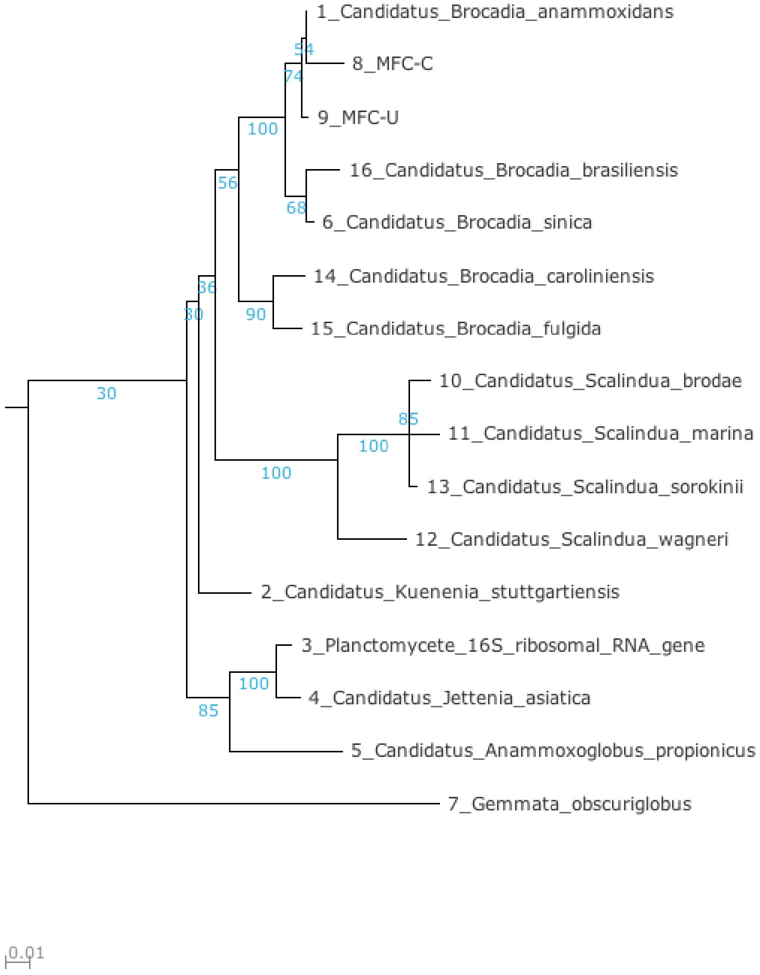
1_Candidatus_Brocadia_anammoxidans
8_MFC-C
9_MFC-U
16_Candidatus_Brocadia_brasiliensis
6_Candidatus_Brocadia_sinica
14_Candidatus_Brocadia_caroliniensis
15_Candidatus_Brocadia_fulgida
10_Candidatus_Scalindua_brodae
11_Candidatus_Scalindua_marina
13_Candidatus_Scalindua_sorokinii
12_Candidatus_Scalindua_wagneri
2_Candidatus_Kuenenia_stuttgartiensis
3_Planctomycete_16S_ribosomal_RNA_gene
4_Candidatus_Jettenia_asiatica
5_Candidatus_Anammoxoglobus_propionicus
7_Gemmata_obscuriglobus
54
74
100
68
56
36
30
90
85
100
100
100
85
30
0.01

Supplementary Figure 2: Unrooted neighbour joining tree based on the partial 16S rRNA gene sequences of the anammox bacteria from MFC-C and MFC-U. 16S rRNA of the anammox bacteria is amplified by a primer set of Brod541F-Brod1260R.
